# Supplementary material for: Intra-Host Evolution Analyses in an Immunosuppressed Patient Supports SARS-CoV-2 Viral Reservoir Hypothesis
Source: Viruses. 2024 Feb 23;16(3):342. doi: 10.3390/v16030342 (PMC10974702; doi:10.3390/v16030342)
Supplement: Supplementary file 1 [file viruses-16-00342-s001.zip › viruses-2868556-supplementary.pdf]

Supplementary Material

Table S1. Description of analyzed samples

ID	Date	Days since symptom onset (DSO)	Tissue
L00409639001	08/01/2021	1	Nasopharyngeal swab
L00350036001A	28/04/2021	111	Nasopharyngeal swab
L00350839	03/05/2021	116	Nasopharyngeal swab
L00363495001	28/06/2021	172	Nasopharyngeal swab

Table S2. Plasma binding and Fc-effector functions of analyzed samples.

	Plasma binding (FACS, normalized to CV3-25)		ADCC (%)
Sample	D614G	B.1.160 (S477N+D614G)	D614G
5/5/2021 (D118)	10,407	19,819	12,11750306
6/5/2021 (D119)	12,367	19,540	20,93023256
10/5/2021 (D123)	7,950	12,850	25,33659731
Threshold	0,985	0,057	7,180742554

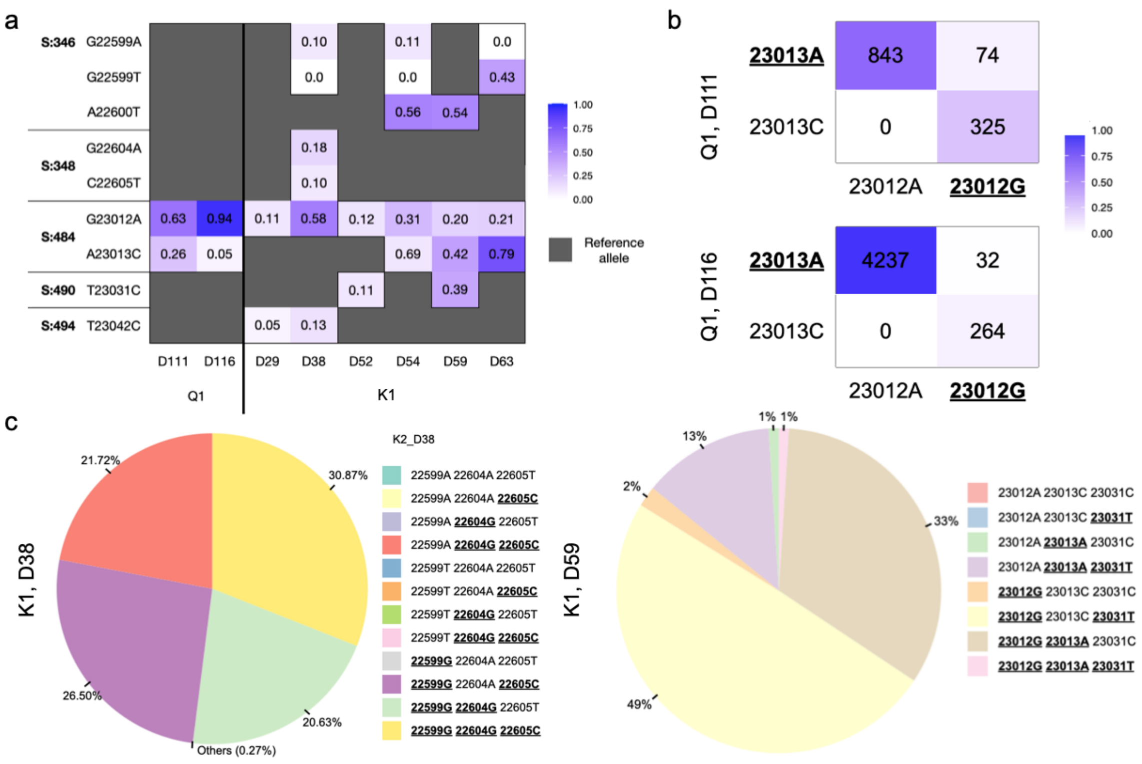

Figure S1. Intra-host allelic frequencies for mutated positions in the S's RBD.

a: frequencies for the alternative allele per position for mutated positions at S:346, S:348, S:484, S:490, and S:494 in the patient described in this study and Lee et al.'s P2 (10). **b**: frequencies of the haplotypes present at codon S:484 on D111 and D116 for our patient. Reference alleles are underlined. **c**: Left - frequencies of the haplotypes present at codons S:346 and S:348 on D38 for P2 on reads encompassing all three positions. "Others" category includes 22599A/22604G/22605T, 22599G/22604A/22605T, and 22599T/22604G/22605C combinations (0.1%, 0.07%, 0.1%, respectively). Right - frequencies for the haplotypes present at codons S:484 and S:490 on D59 for P2 on reads containing all three positions.
